# Erysipelas, Diphtheria, and Scarlatina

**Published:** 1863-10

**Authors:** A. Fisher

**Affiliations:** Chicago, Ill.


ARTICLE XXVI.

### ERYSIPELAS, DIPHTHERIA, AND SCARLATINA.

BY A. FISHER, M.D., of Chicago, Ill.

Sanitary Report read before the Chicago Medical Society, August 4th.

In reporting the sanitary condition of the South Division for the past three months, there is little to say. As far as I can learn, there has been no epidemic nor any unusual amount of disease of any kind. On the contrary, I think the city may now be regarded as uncommonly healthy, with fewer cases of bowel-complaints of children than we generally have at this season of the year, at least I find it so in my practice, and the physicians with whom I have conversed on the subject say, they have not had as many cases of the kind this summer as usual; besides, what few cases I have had have been very easily controlled, without the disposition to relapse, which is so common some seasons. However, within a short time I have had two cases of acute enteritis and one case of acute gastro-enteritis of adult females, which were very severe. The disease was controlled, in all of them, in a few days by cupping and leeching freely, with applications of hot fomentations of hops applied over the stomach and bowels, and continued for two or three days, renewing them as often as they became cold, together with ¼ gr. doses of calomel rubbed up with sugar and given every hour, and continued until the vomiting and great thirst was relieved and the secretions restored. I mention these cases because they are so very rare in my practice that I do not remember of having a well-marked case of the kind before for the past year, and to ascertain whether other members of the Society have met with many such cases this summer or whether they were accidental.

Typhoid fever has not prevailed to any extent during the summer, to my knowledge; and I cannot learn that bilious, remittent, or intermittent fever has been very prevalent, though, as usual, there are cases of the kind. There has been more or less whooping-cough during the summer, perhaps more than usual, but not of a serious character.

I have not seen or heard of a case of measles this summer, still there may and, probably, have been cases which have not come to my knowledge.

The variola and varioloid which was so prevalent here in the winter and spring has nearly subsided, though I understand there has been quite a number of cases during the summer, and even now a case occasionally occurs, showing that the virus still remains among us. When we find many children, and occasionally an adult, who has not been properly vaccinated, it is not strange that the disease should continue to be propagated in a city like this. The wonder is, that it should ever be eradicated so long as there are so many who have not been protected by vaccination. In my opinion, there should be some way devised that every adult and child, over four or six months old, should in some way be compelled to be properly vaccinated. In that way only can we be sure that the pestilence will be entirely eradicated and kept from the city.

Erysipelas, scarlatina, and diphtheria, which prevailed to some extent last spring, I believe have nearly subsided, although a sporadic case occasionally occurs this summer. In the spring, quite a number of cases of erysipelas occurred in my practice, and among them four very interesting cases in one family, which I intended to report long ago, but as this is my first Sanitary Report, I will give a synopsis of them now, to show the intimate relation between diphtheria, scarlatina, and erysipelas:—

Mr. D.'s family consisted of himself, wife, and two children, one about three and the other about five years old. Whilst I was treating Mrs. D. for diphtheria, in March last, Mr. D. was taken with the same disease. His pulse was frequent and weak; throat badly swollen, with the characteristic ash-colored specks and patches of false membrane; he was very weak and prostrated in a short time after the commencement of the disease. I treated the case with chlorate potassa acidulated with hydrochloric acid, with sul. quinine, tinc. ferri murias, &c. In about a week the disease subsided, and he began to take nourishment with a relish, when, suddenly, erysipelas commenced on both ears, and spread over the cheeks, but, by pencilling it with nitrate of silver, it was so far arrested that it did not spread over the face and forehead, though it continued over the scalp. Prescribed ʒss. tinc. ferri murias every four hours, and continued it for four or five days, when the inflammation subsided and he began to recover. Then the right side of his face and neck commenced swelling, and continued to increase until the right tonsil, tongue, and adjacent parts were so much swollen that it was with great difficulty that he could swallow. The inflammation was, in its character, diffuse, with infiltration into the cellular tissue, hard and unyielding, without any signs of pointing, either externally or internally, Under the circumstances, I proposed counsel, and Dr. DAVIS was selected; but, just before he arrived, hemorrhage suddenly commenced from the right ear, when they sent for me immediately, and before I saw him, which was not more than ten or fifteen minutes after the commencement of the hemorrhage, he had lost at least a quart of blood, and was apparently sinking in collapse when Dr. DAVIS arrived. The loss of blood prostrated him very much, and did not in the least diminish the swelling.

My treatment had been tinc. ferri murias with chlorate potassa and quinine as a tonic, and, for a few hours before the arrival of Dr. DAVIS, had been trying to give him in addition sulphite soda, but, owing to the great difficulty of swallowing, did not get down much of anything. Dr. DAVIS recommended a perseverance in the remedies as the only hope. He continued in a collapsed condition for about forty-eight hours and died. We could not obtain the privilege of making a *post mortem* examination, which I very much regret.

How to account for the loss of such a quantity of blood in so short a time I cannot imagine. It was arterial blood, and was poured out in a full stream so that it was caught in a wash-bowl. As before stated, it could not have been more than ten or fifteen minutes after he commenced bleeding before I saw him. I immediately plugged the ear with cotton and arrested the hemorrhage, and it did not return. I know of no artery in the internal ear large enough to discharge such a quantity of blood in so short a time, even if it were severed.

Whilst Mr. D. was sick, both of his children were taken at the same time. The one five years old, with a swelled throat; weak and frequent pulse; with stupor. The other, about three years old, with a scarlet rash, covering every part of the surface, with erysipelas of the inferior extremities. Her pulse was frequent; tongue red; and throat sore, without much swelling. As I was leaving the house the next day, after she was taken, I met Dr. HATCH at the door and requested him to diagnose the case. When he first saw her, he said, it was a well-marked case of scarlatina. After exposing her extremities, he said, it was scarlatina complicated with erysipelas. The treatment of these two cases was the same, viz., one drachm of a saturated solution of sulphite of soda every two hours, continued without intermission for five days, with no other remedy. After taking the sulphite of soda for forty-eight hours, the constitutional symptoms in both cases were entirely relieved, and the swelled throat of the oldest began to subside. The rash and erysipelas of the youngest remained the usual time, and was followed by the ordinary disquamation, though her appetite was good; and the constitutional symptoms subsided the third day, and convalescence was rapid and permanent.

We have here four cases of zymotic disease in one family, at the same time, effecting every member of it differently, viz., one with diphtheria alone; one with diphtheria, followed by erysipelas of the face and head, and diffuse inflammation of the side of the neck; one with scarlatina, with erysipelas of the inferior extremities; and the other, in a stupid condition, with a swelled throat.

Now, it is evident to me that all of these cases originated from the same abnormal condition of the blood; for although there was a material difference in the manifestation, appearance, and location of the disease in the different cases, the constitutional symptoms were very much alike,—all showing a depression of the vital powers, with other symptoms, such as we have in catalytic or blood-poison diseases, which any physician of experience will surely detect.

Whether the poison which causes these various diseases is the same, generating the same morbid condition of the blood in diphtheria, scarlatina, erysipelas, and other zymotic diseases; or whether there is a specific poison for each of those diseases is a question of importance yet to be solved. The pathological condition of the blood in the so-called blood-poison diseases is attracting the attention of pathologists, both in this country and Europe; and I trust the time is not far distant when we shall understand the nature of such cases better, and be able to treat them in a more scientific manner.

Now, who will not say, that these cases, though differently manifested, originated from the same abnormal condition of the blood? I believe it most sincerely, and that the same constitutional treatment would be applicable to all; and were I to meet with such a group of cases again, I would give the sulphites to all of them, beginning early, and continue them with such other remedies as seemed to be indicated for the local disease.

